# Supplementary material for: Modeling glioblastoma heterogeneity as a dynamic network of cell states
Source: Mol Syst Biol. 2021 Sep 16;17(9):e10105. doi: 10.15252/msb.202010105 (PMC8444284; doi:10.15252/msb.202010105)
Supplement: Supplementary file 5 — Source Data for Figure 3 [file MSB-17-e10105-s001.zip › Figure3A_sourcedata/GSEA_3065/hallmarks_state2.GseaPreranked.1623416269049/HALLMARK_MTORC1_SIGNALING.html]

Details for gene set HALLMARK\_MTORC1\_SIGNALING[GSEA]

|  || Dataset | state2 |
| Phenotype | NoPhenotypeAvailable |
| Upregulated in class | na\_pos |
| GeneSet | HALLMARK\_MTORC1\_SIGNALING |
| Enrichment Score (ES) | 0.3535148 |
| Normalized Enrichment Score (NES) | 1.2630391 |
| Nominal p-value | 0.07177033 |
| FDR q-value | 0.20004278 |
| FWER p-Value | 0.982 |
Table: GSEA Results Summary

  

Fig 1: Enrichment plot: HALLMARK\_MTORC1\_SIGNALING      
 Profile of the Running ES Score & Positions of GeneSet Members on the Rank Ordered List

  

| PROBE | GENE SYMBOL | GENE\_TITLE | RANK IN GENE LIST | RANK METRIC SCORE | RUNNING ES | CORE ENRICHMENT || 1 | DHFR |  |  | 11 | 0.644 | 0.0296 | Yes |
| 2 | RRM2 |  |  | 22 | 0.556 | 0.0550 | Yes |
| 3 | MCM4 |  |  | 79 | 0.399 | 0.0680 | Yes |
| 4 | CACYBP |  |  | 109 | 0.366 | 0.0824 | Yes |
| 5 | HSPD1 |  |  | 176 | 0.294 | 0.0893 | Yes |
| 6 | DDX39A |  |  | 183 | 0.293 | 0.1026 | Yes |
| 7 | PPIA |  |  | 189 | 0.287 | 0.1158 | Yes |
| 8 | HSPE1 |  |  | 211 | 0.276 | 0.1267 | Yes |
| 9 | MCM2 |  |  | 228 | 0.262 | 0.1375 | Yes |
| 10 | TUBG1 |  |  | 262 | 0.246 | 0.1456 | Yes |
| 11 | PHGDH |  |  | 271 | 0.243 | 0.1564 | Yes |
| 12 | TPI1 |  |  | 301 | 0.231 | 0.1643 | Yes |
| 13 | CCT6A |  |  | 314 | 0.227 | 0.1739 | Yes |
| 14 | TMEM97 |  |  | 331 | 0.223 | 0.1828 | Yes |
| 15 | BUB1 |  |  | 335 | 0.222 | 0.1930 | Yes |
| 16 | PLK1 |  |  | 364 | 0.213 | 0.2002 | Yes |
| 17 | AURKA |  |  | 375 | 0.210 | 0.2091 | Yes |
| 18 | UNG |  |  | 398 | 0.200 | 0.2163 | Yes |
| 19 | GMPS |  |  | 420 | 0.195 | 0.2234 | Yes |
| 20 | PSMG1 |  |  | 428 | 0.193 | 0.2319 | Yes |
| 21 | PDAP1 |  |  | 454 | 0.190 | 0.2382 | Yes |
| 22 | LDHA |  |  | 506 | 0.179 | 0.2413 | Yes |
| 23 | UCHL5 |  |  | 538 | 0.174 | 0.2463 | Yes |
| 24 | TOMM40 |  |  | 560 | 0.171 | 0.2521 | Yes |
| 25 | PSAT1 |  |  | 561 | 0.170 | 0.2603 | Yes |
| 26 | HPRT1 |  |  | 573 | 0.168 | 0.2671 | Yes |
| 27 | CDC25A |  |  | 578 | 0.167 | 0.2746 | Yes |
| 28 | RPA1 |  |  | 687 | 0.151 | 0.2702 | Yes |
| 29 | ENO1 |  |  | 717 | 0.148 | 0.2741 | Yes |
| 30 | PGK1 |  |  | 726 | 0.146 | 0.2802 | Yes |
| 31 | ARPC5L |  |  | 767 | 0.142 | 0.2827 | Yes |
| 32 | STIP1 |  |  | 794 | 0.139 | 0.2865 | Yes |
| 33 | PSMA4 |  |  | 797 | 0.138 | 0.2929 | Yes |
| 34 | UBE2D3 |  |  | 820 | 0.136 | 0.2970 | Yes |
| 35 | PSMC2 |  |  | 858 | 0.132 | 0.2993 | Yes |
| 36 | PSMA3 |  |  | 866 | 0.131 | 0.3048 | Yes |
| 37 | IDI1 |  |  | 878 | 0.130 | 0.3098 | Yes |
| 38 | CCNF |  |  | 895 | 0.128 | 0.3142 | Yes |
| 39 | EEF1E1 |  |  | 903 | 0.128 | 0.3196 | Yes |
| 40 | GAPDH |  |  | 933 | 0.125 | 0.3224 | Yes |
| 41 | ATP5MC1 |  |  | 935 | 0.125 | 0.3283 | Yes |
| 42 | IMMT |  |  | 988 | 0.121 | 0.3284 | Yes |
| 43 | HSPA9 |  |  | 996 | 0.120 | 0.3334 | Yes |
| 44 | MTHFD2 |  |  | 1014 | 0.118 | 0.3372 | Yes |
| 45 | PPA1 |  |  | 1052 | 0.115 | 0.3387 | Yes |
| 46 | NUP205 |  |  | 1147 | 0.108 | 0.3338 | Yes |
| 47 | PSMB5 |  |  | 1168 | 0.106 | 0.3367 | Yes |
| 48 | PSMC4 |  |  | 1237 | 0.102 | 0.3342 | Yes |
| 49 | EIF2S2 |  |  | 1245 | 0.102 | 0.3383 | Yes |
| 50 | TCEA1 |  |  | 1262 | 0.101 | 0.3414 | Yes |
| 51 | COPS5 |  |  | 1302 | 0.098 | 0.3419 | Yes |
| 52 | PNO1 |  |  | 1351 | 0.095 | 0.3412 | Yes |
| 53 | HMBS |  |  | 1393 | 0.092 | 0.3412 | Yes |
| 54 | PSMD12 |  |  | 1411 | 0.092 | 0.3437 | Yes |
| 55 | SHMT2 |  |  | 1449 | 0.090 | 0.3440 | Yes |
| 56 | BCAT1 |  |  | 1503 | 0.086 | 0.3424 | Yes |
| 57 | ACTR3 |  |  | 1528 | 0.085 | 0.3439 | Yes |
| 58 | PSMD13 |  |  | 1532 | 0.085 | 0.3476 | Yes |
| 59 | RRP9 |  |  | 1583 | 0.082 | 0.3461 | Yes |
| 60 | RAB1A |  |  | 1609 | 0.081 | 0.3473 | Yes |
| 61 | PSMD14 |  |  | 1627 | 0.080 | 0.3493 | Yes |
| 62 | PSME3 |  |  | 1706 | 0.077 | 0.3446 | Yes |
| 63 | SERP1 |  |  | 1717 | 0.076 | 0.3471 | Yes |
| 64 | GSR |  |  | 1739 | 0.075 | 0.3484 | Yes |
| 65 | ETF1 |  |  | 1749 | 0.075 | 0.3510 | Yes |
| 66 | PRDX1 |  |  | 1763 | 0.074 | 0.3532 | Yes |
| 67 | PITPNB |  |  | 1793 | 0.073 | 0.3535 | Yes |
| 68 | CYB5B |  |  | 1850 | 0.070 | 0.3508 | No |
| 69 | PSMC6 |  |  | 1874 | 0.070 | 0.3517 | No |
| 70 | HSPA4 |  |  | 2227 | 0.056 | 0.3163 | No |
| 71 | QDPR |  |  | 2314 | 0.053 | 0.3096 | No |
| 72 | GPI |  |  | 2330 | 0.053 | 0.3105 | No |
| 73 | ADD3 |  |  | 2401 | 0.051 | 0.3054 | No |
| 74 | PFKL |  |  | 2489 | 0.048 | 0.2983 | No |
| 75 | IFRD1 |  |  | 2840 | 0.038 | 0.2623 | No |
| 76 | NFYC |  |  | 2889 | 0.037 | 0.2588 | No |
| 77 | GGA2 |  |  | 2961 | 0.035 | 0.2528 | No |
| 78 | ACTR2 |  |  | 3044 | 0.033 | 0.2456 | No |
| 79 | SORD |  |  | 3056 | 0.033 | 0.2459 | No |
| 80 | POLR3G |  |  | 3472 | 0.024 | 0.2023 | No |
| 81 | MAP2K3 |  |  | 3478 | 0.024 | 0.2029 | No |
| 82 | NFKBIB |  |  | 3488 | 0.024 | 0.2031 | No |
| 83 | CORO1A |  |  | 3519 | 0.024 | 0.2010 | No |
| 84 | ADIPOR2 |  |  | 3549 | 0.023 | 0.1989 | No |
| 85 | GOT1 |  |  | 3561 | 0.023 | 0.1988 | No |
| 86 | SRD5A1 |  |  | 3809 | 0.019 | 0.1730 | No |
| 87 | SLC9A3R1 |  |  | 3823 | 0.018 | 0.1725 | No |
| 88 | SDF2L1 |  |  | 3885 | 0.017 | 0.1668 | No |
| 89 | PGM1 |  |  | 3936 | 0.016 | 0.1622 | No |
| 90 | FADS1 |  |  | 3941 | 0.016 | 0.1625 | No |
| 91 | NUFIP1 |  |  | 3950 | 0.016 | 0.1624 | No |
| 92 | SLC37A4 |  |  | 3957 | 0.016 | 0.1626 | No |
| 93 | GCLC |  |  | 4056 | 0.015 | 0.1527 | No |
| 94 | NMT1 |  |  | 4164 | 0.013 | 0.1417 | No |
| 95 | NFIL3 |  |  | 4213 | 0.012 | 0.1371 | No |
| 96 | HMGCS1 |  |  | 4272 | 0.011 | 0.1314 | No |
| 97 | LTA4H |  |  | 4370 | 0.009 | 0.1213 | No |
| 98 | GLA |  |  | 4388 | 0.009 | 0.1199 | No |
| 99 | ALDOA |  |  | 4435 | 0.008 | 0.1154 | No |
| 100 | NAMPT |  |  | 4573 | 0.006 | 0.1009 | No |
| 101 | MLLT11 |  |  | 4607 | 0.006 | 0.0976 | No |
| 102 | ATP6V1D |  |  | 4650 | 0.005 | 0.0933 | No |
| 103 | YKT6 |  |  | 4828 | 0.002 | 0.0743 | No |
| 104 | CCNG1 |  |  | 4843 | 0.002 | 0.0728 | No |
| 105 | STARD4 |  |  | 5079 | -0.001 | 0.0475 | No |
| 106 | SYTL2 |  |  | 5278 | -0.005 | 0.0264 | No |
| 107 | ERO1A |  |  | 5380 | -0.006 | 0.0158 | No |
| 108 | UFM1 |  |  | 5435 | -0.007 | 0.0103 | No |
| 109 | ASNS |  |  | 5539 | -0.009 | -0.0004 | No |
| 110 | SKAP2 |  |  | 5558 | -0.009 | -0.0019 | No |
| 111 | SLC1A5 |  |  | 5584 | -0.010 | -0.0041 | No |
| 112 | G6PD |  |  | 5598 | -0.010 | -0.0051 | No |
| 113 | GTF2H1 |  |  | 5636 | -0.011 | -0.0086 | No |
| 114 | INSIG1 |  |  | 5815 | -0.013 | -0.0271 | No |
| 115 | EBP |  |  | 6195 | -0.020 | -0.0671 | No |
| 116 | IDH1 |  |  | 6208 | -0.020 | -0.0675 | No |
| 117 | ACACA |  |  | 6253 | -0.021 | -0.0712 | No |
| 118 | MTHFD2L |  |  | 6324 | -0.022 | -0.0777 | No |
| 119 | GSK3B |  |  | 6331 | -0.022 | -0.0773 | No |
| 120 | TXNRD1 |  |  | 6419 | -0.024 | -0.0856 | No |
| 121 | DDIT3 |  |  | 6433 | -0.024 | -0.0858 | No |
| 122 | LGMN |  |  | 6457 | -0.025 | -0.0871 | No |
| 123 | TBK1 |  |  | 6762 | -0.031 | -0.1185 | No |
| 124 | GBE1 |  |  | 6821 | -0.032 | -0.1232 | No |
| 125 | XBP1 |  |  | 6931 | -0.035 | -0.1333 | No |
| 126 | PPP1R15A |  |  | 7004 | -0.037 | -0.1393 | No |
| 127 | ELOVL5 |  |  | 7020 | -0.037 | -0.1392 | No |
| 128 | PSPH |  |  | 7132 | -0.040 | -0.1493 | No |
| 129 | DHCR24 |  |  | 7171 | -0.041 | -0.1514 | No |
| 130 | SLC1A4 |  |  | 7280 | -0.044 | -0.1609 | No |
| 131 | ACLY |  |  | 7320 | -0.045 | -0.1630 | No |
| 132 | CYP51A1 |  |  | 7467 | -0.050 | -0.1764 | No |
| 133 | EDEM1 |  |  | 7566 | -0.052 | -0.1845 | No |
| 134 | FDXR |  |  | 7726 | -0.057 | -0.1989 | No |
| 135 | RPN1 |  |  | 7869 | -0.062 | -0.2113 | No |
| 136 | RIT1 |  |  | 7880 | -0.062 | -0.2094 | No |
| 137 | SC5D |  |  | 7900 | -0.063 | -0.2085 | No |
| 138 | SLC7A5 |  |  | 8067 | -0.070 | -0.2230 | No |
| 139 | USO1 |  |  | 8086 | -0.071 | -0.2216 | No |
| 140 | GLRX |  |  | 8100 | -0.071 | -0.2196 | No |
| 141 | SLC6A6 |  |  | 8167 | -0.075 | -0.2231 | No |
| 142 | M6PR |  |  | 8183 | -0.076 | -0.2211 | No |
| 143 | VLDLR |  |  | 8186 | -0.077 | -0.2177 | No |
| 144 | P4HA1 |  |  | 8398 | -0.089 | -0.2362 | No |
| 145 | PLOD2 |  |  | 8403 | -0.089 | -0.2324 | No |
| 146 | SLC2A3 |  |  | 8419 | -0.090 | -0.2297 | No |
| 147 | FADS2 |  |  | 8492 | -0.095 | -0.2329 | No |
| 148 | SEC11A |  |  | 8575 | -0.101 | -0.2369 | No |
| 149 | DDIT4 |  |  | 8601 | -0.103 | -0.2347 | No |
| 150 | TFRC |  |  | 8650 | -0.107 | -0.2348 | No |
| 151 | SSR1 |  |  | 8765 | -0.118 | -0.2414 | No |
| 152 | SQSTM1 |  |  | 8790 | -0.121 | -0.2383 | No |
| 153 | BHLHE40 |  |  | 8804 | -0.123 | -0.2338 | No |
| 154 | PIK3R3 |  |  | 8840 | -0.127 | -0.2315 | No |
| 155 | FKBP2 |  |  | 8892 | -0.134 | -0.2306 | No |
| 156 | ATP2A2 |  |  | 8946 | -0.141 | -0.2296 | No |
| 157 | SCD |  |  | 8982 | -0.146 | -0.2264 | No |
| 158 | DHCR7 |  |  | 9009 | -0.150 | -0.2221 | No |
| 159 | SLC7A11 |  |  | 9040 | -0.156 | -0.2179 | No |
| 160 | SERPINH1 |  |  | 9054 | -0.159 | -0.2117 | No |
| 161 | ELOVL6 |  |  | 9070 | -0.161 | -0.2056 | No |
| 162 | RDH11 |  |  | 9108 | -0.167 | -0.2016 | No |
| 163 | LDLR |  |  | 9117 | -0.170 | -0.1944 | No |
| 164 | ACSL3 |  |  | 9175 | -0.184 | -0.1918 | No |
| 165 | HMGCR |  |  | 9194 | -0.187 | -0.1848 | No |
| 166 | SQLE |  |  | 9209 | -0.194 | -0.1770 | No |
| 167 | BTG2 |  |  | 9239 | -0.207 | -0.1703 | No |
| 168 | CDKN1A |  |  | 9301 | -0.230 | -0.1659 | No |
| 169 | CALR |  |  | 9310 | -0.237 | -0.1554 | No |
| 170 | CD9 |  |  | 9324 | -0.248 | -0.1450 | No |
| 171 | CXCR4 |  |  | 9328 | -0.251 | -0.1333 | No |
| 172 | TM7SF2 |  |  | 9330 | -0.252 | -0.1214 | No |
| 173 | HSP90B1 |  |  | 9334 | -0.257 | -0.1095 | No |
| 174 | SLC2A1 |  |  | 9349 | -0.272 | -0.0980 | No |
| 175 | CANX |  |  | 9374 | -0.310 | -0.0858 | No |
| 176 | STC1 |  |  | 9378 | -0.314 | -0.0711 | No |
| 177 | HSPA5 |  |  | 9406 | -0.395 | -0.0552 | No |
| 178 | CTSC |  |  | 9413 | -0.431 | -0.0352 | No |
| 179 | IGFBP5 |  |  | 9438 | -0.797 | 0.0002 | No |
Table: GSEA details [plain text format]

  

Fig 2: HALLMARK\_MTORC1\_SIGNALING: Random ES distribution      
 Gene set null distribution of ES for **HALLMARK\_MTORC1\_SIGNALING**
